# PDF Entity Annotation Tool (PEAT)

**DOI:** 10.21105/joss.05336

**Published:** 2025-04-08

**Authors:** Christopher G. Stahl, Kristan J. Markey, Brian C. Jewell, Dahnish Shams, Michele M. Taylor, A. Amina Wilkins, Sean Watford, Andy Shapiro, Michelle Angrish

**Affiliations:** 1 Oak Ridge National Laboratory, USA; 2 Office of Research and Development. United States Environmental Protection Agency

## Abstract

While different text mining approaches – including the use of Artificial Intelligence (AI) and other machine based methods - continue to expand at a rapid pace, the tools used by researchers to create the labeled datasets required for training, modeling, and evaluation remain rudimentary. Labeled datasets contain the target attributes the machine is going to learn; for example, training an algorithm to delineate between images of a car or truck would generally require a set of images with a quantitative description of the underlying features of each vehicle type. Development of labeled textual data that can be used to build natural language machine learning models for scientific literature is not currently integrated into existing manual workflows used by domain experts. Published literature is rich with important information, such as different types of embedded text, plots, and tables that can all be used as inputs to train ML/natural language processing (NLP) models, when extracted and prepared in machine readable formats. Currently, both normalized data extraction of use to domain experts and extraction to support development of ML/NLP models are labor intensive and cumbersome manual processes. Automatic extraction of data and information from formats such as PDFs that are optimized for layout and human readability, not machine readability. The PDF (Portable Document Format) Entity Annotation Tool (PEAT) was developed with the goal of allowing users to annotate publications within their current print format, while also allowing those annotations to be captured in a machine-readable format. One of the main issues with traditional annotation tools is that they require transforming the PDF into plain text to facilitate the annotation process. While doing so lessens the technical challenges of annotating data, the user loses all structure and provenance that was inherent in the underlying PDF. Also, textual data extraction from PDFs can be an error prone process. Challenges include identifying sequential blocks of text and a multitude of document formats (multiple columns, font encodings, etc.). As a result of these challenges, using existing tools for development of NLP/ML models directly from PDFs is difficult because the generated outputs are not interoperable.

We created a system that allows annotations to be completed on the original PDF document structure, with no plain text extraction. The result is an application that allows for easier and more accurate annotations. In addition, by including a feature that grants the user the ability to easily create a schema, we have developed a system that can be used to annotate text for different domain-centric schemas of relevance to subject matter experts. Different knowledge domains require distinct schemas and annotation tags to support machine learning.

## Statement of need

Data users are confronted with new data being published faster than manual data extractions are practical. Millions of publications are produced every year (White, 2021), potentially containing useful information for ongoing and future research, experiments, etc. With the sheer amount of data needing to be processed, it is unsustainable to manually keep up with the information being produced. The development and application of ML tools to help process and manage useful information from these publications is critically important to ensure that the most recent evidence is identified, evaluated, and extracted. Development of ML models requires manually labelled datasets, but currently, generation of those datasets is a high level of effort endeavor and not aligned with existing workflows used to manually summarize content presented in full-text (e.g., literature review workflows). Scientific literature is almost exclusively made available in print publications that are locked behind non-machine-readable formats, primarily PDF. The PDF format was designed to create digital versions of paper documents while maintaining their visual look and structure. While the format handles that task very well, it does not lend to the extraction of data to other formats. The opening of the PDF standard in 2008 (International Organization for Standardization, 2020) has helped facilitate PDF text extraction, but document formatting and content (e.g., coordinates of text within a document) can still be lost during PDF to text conversion using currently available tools (Xu et al., 2013).

Existing annotation solutions such as BRAT (Stenetorp et al., 2012), TeamTat (Islamaj et al., 2020), GATE (*GATE*, 2022), and Dextr (Walker et al., 2022) rely on first converting the PDF to a plain text layer before annotation by the user. Not only does this require the researcher to annotate unstructured plain text as opposed to a fully formatted PDF document, but also in many instances the PDF’s format (double/triple columns, tables, etc) would fail to extract and render a document unusable. This is due to the nature of the PDF document creation process. Text can be split into several chunks (splits within words, lines, columns, etc.) and extraction tools use heuristics to attempt to put the chunks back together like a puzzle. A separate tool, PDFAnno (Shindo et al., 2018), improved on this by allowing annotation layers to be created on top of PDF documents but is no longer maintained or functional. Reac-PDF Annot (Tyurin, 2022) is a library that allows for basic annotations to be created in on top of PDFJS (Mozilla, 2025) based applications and was used as a baseline for our application.

**Figure 1:**
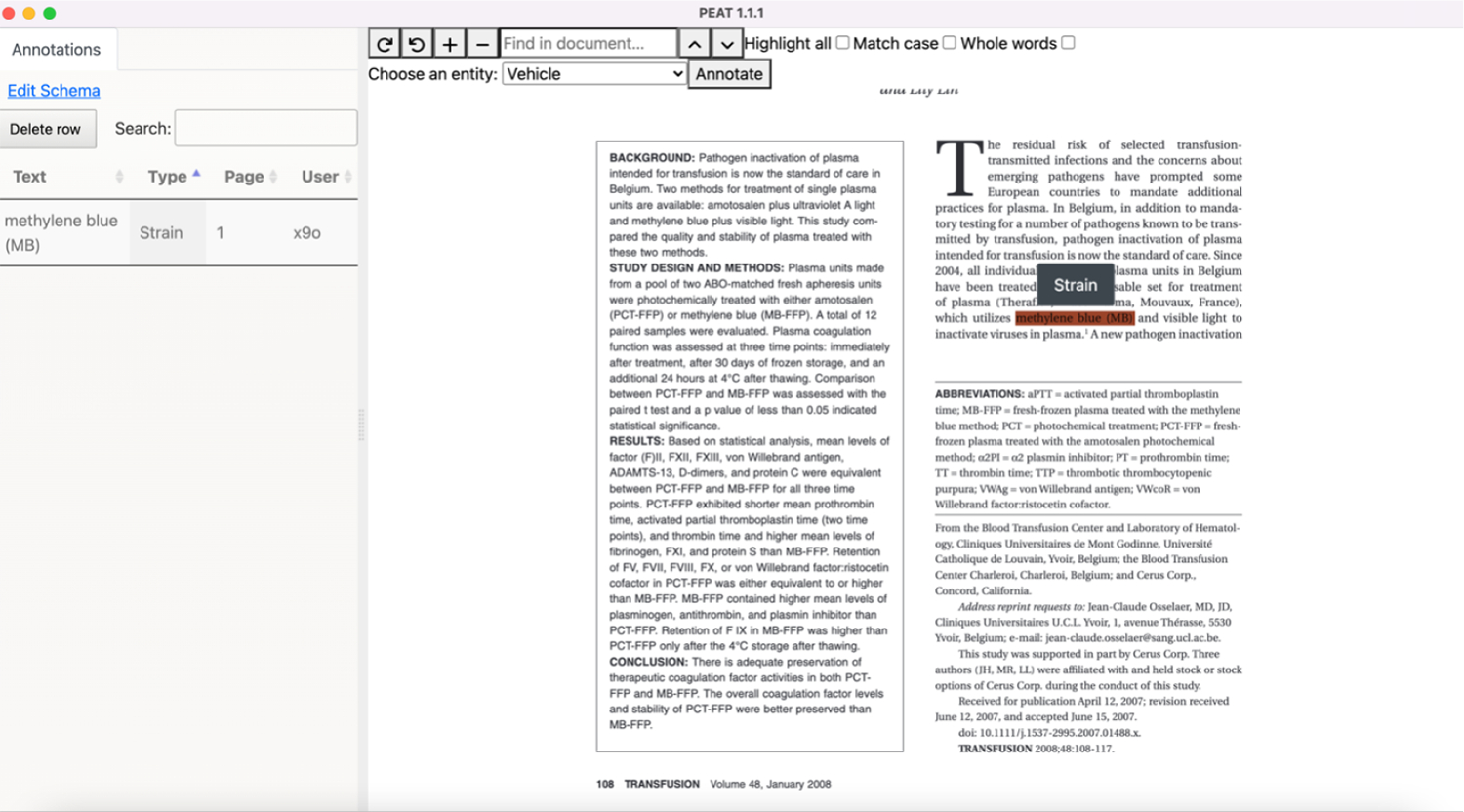
PEAT User Interface.

PEAT was designed to take advantage of the latest advancements in PDF text extraction methods while also allowing the user to annotate and label the data directly in PDF format. This approach allows a user to work in a document structure they are familiar with, improving the user experience and facilitating the creation of labeled data for machine consumption and training of future machine learning models. PEAT is a portable, standalone application built off the Electron software framework (Electron Authors, 2025) and can be used on all major operating systems (Windows, Linux, and Macintosh) and provides an interface for users to annotate PDFs that are displayed in their intended format (Figure 1).

The portability is accomplished by embedding all necessary dependencies into the framework, which does result in a larger than usual footprint, PEAT is 500 MB. The application allows users to load PDFs directly from their file system along with data annotation forms with standard or customizable annotation types, labels, entities, and other features such as custom color highlighting. The application also includes features for users to edit and import/export data extraction schemas, export annotations of X and Y PDF coordinate structure (based on the image layer of the PDF), search and manipulate annotations, and save/load progress. Once a user has completed document annotation, the labeled data, full text, and all associated metadata is exportable in JSON format that can be processed by a variety of NLP model building applications such as Spacy or PyTorch (Ansel et al., 2024; Honnibal et al., 2020).

## Conclusion and Future Work

In this work we demonstrated PEAT’s ability to assist users in the creation of annotated datasets that provide a machine-readable output suitable for machine learning applications. It allows users to annotate PDF documents in their native form using standard or custom annotations suitable for direct use with named entity recognition tools. Additionally, the schema editor tool grants users the flexibility to customize their annotations for domain specific applications. PEAT provides the foundation for many additional features such as collaborative annotation, a hierarchical annotation system (i.e., groups of annotations forming “relationships”), auto annotation based on imported ontologies, and more. Finally, creating a pluggable architecture for generating and aligning text layers or segments which would be used for annotations and NLP processing would be desirable. These feature enhancements would further increase the user’s ability to create more advanced annotation sets, laying the groundwork for the continued growth and evolution of machine learning applications.
